# The relaxation exercise and social support trial-resst: study protocol for a randomized community based trial

**DOI:** 10.1186/1471-244X-11-142

**Published:** 2011-08-25

**Authors:** Loulou Kobeissi, Ricardo Araya, Fayssal El Kak, Zeina Ghantous, Marwan Khawaja, Brigitte Khoury, Ziyad Mahfoud, Rima Nakkash, Tim J Peters, Sami Ramia, Huda Zurayk

**Affiliations:** 1 UCLA, School of Public Health, Community Health Sciences Department, 650 E Charles Young Dr, Los Angeles, California 90095, USA; 2 Center for Research on Population and Health, Epidemiology and Population Health Department, Faculty of Health Sciences, American University of Beirut, Beirut, Lebanon; 3 Academic Unit of Psychiatry, School of Social and Community Medicine, University of Bristol, Bristol, UK; 4 Department of Health Promotion and Community Health, Faculty of Health Sciences, American University of Beirut, Beirut, Lebanon; 5 Department of Psychiatry, Faculty of Medicine, American University of Beirut, Beirut, Lebanon; 6 Department of Public Health, Weill Cornell Medical College, Doha, Qatar; 7 Center for Research on Population and Health, Department of Health Promotion and Community Health, Faculty of Health Sciences, American University of Beirut, Beirut, Lebanon; 8 School of Clinical Sciences, University of Bristol, Bristol, UK; 9 Medical Lab Sciences Program, Faculty of Health Sciences, American University of Beirut, Beirut, Lebanon

## Abstract

**Background:**

Studies suggests a possible link between vaginal discharge and common mental distress, as well as highlight the implications of the subjective burden of disease and its link with mental health.

**Methods/Design:**

This is a community-based intervention trial that aims to evaluate the impact of a psycho-social intervention on medically unexplained vaginal discharge (MUVD) in a group of married, low-income Lebanese women, aged 18-49, and suffering from low to moderate levels of anxiety and/or depression. The intervention consisted of 12 sessions of structured social support, problem solving techniques, group discussions and trainer-supervised relaxation exercises (twice per week over six weeks). Women were recruited from Hey el Selloum, a southern disadvantaged suburb of Beirut, Lebanon, during an open recruitment campaign. The primary outcome was self-reported MUVD, upon ruling out reproductive tract infections (RTIs), through lab analysis. Anxiety and/or depression symptoms were the secondary outcomes for this trial. These were assessed using an Arabic validated version of the Hopkins Symptoms Checklist-25 (HSCL-25). Assessments were done at baseline and six months using face-to face interviews, pelvic examinations and laboratory tests. Women were randomized into either intervention or control group. Intent to treat analysis will be used.

**Discussion:**

The results will indicate whether the proposed psychosocial intervention was effective in reducing MUVD (possibly mediated by common mental distress).

**Trial Registration:**

The trial is registered at the Wellcome Trust Registry, ISRCTN assigned: ISRCTN: ISRCTN98441241

## Background

The Urban Health Study (UHS) undertaken in 2002-2003 provided the baseline survey data for the proposed intervention in Hey el Sellom. The UHS sample consisted of 3,100 households from three communities (Nabaa, Burj el Barajneh and Hay el Selloum) located in Beirut's poor and rapidly changing suburbs. All ever-married women (n = 1899) found in these selected households were subsequently interviewed to provide data on the social context of health, among which reproductive health.

The UHS findings indicated that abnormal vaginal discharge was a common complaint in Hey el Selloum. 38% of ever-married women aged 15-59 years in Hey el Sellom [1] complained of vaginal discharge, of whom 71% reported being 'bothered' by the complaint. Of those reporting vaginal discharge, 64% already consulted a health provider or planned to do so, of whom only a few actually reported having reproductive tract infections.

It has been estimated that more than half of the women in the Arab world suffer from symptoms associated with reproductive health problems; and the most commonly reported symptom is vaginal discharge [2-4]. Increasing evidence, on the other hand, indicates the poor association between reported symptoms of reproductive health problems and medically diagnosed diseases [5-9]. Vaginal discharge, being the most common reported gynaecological symptom of RTIs, has recently received more attention [1,5,10-12]. Patel V. *et al*., (2005), in a population-based survey conducted in Goa, India, showed the lack of association between complaints of vaginal discharge and the presence of medically diagnosed reproductive tract infections (RTIs). In another community-based study, conducted in three villages of Giza in Egypt, a similar finding was also observed. 77% of interviewed women in the study reported abnormal vaginal discharge, among whom 52% actually suffered from the presence of one or more RTIs [5]. In addition to the UHS, a study in Lebanon [6] conducted in Nebi Shite- Lebanese community, which shares similar socio-demographic characteristics to Hey el Sellom, showed similar observations. 24.5% of the women in the Nebi Shite survey reported vaginal discharge, while only 9.3% of these women actually suffered from RTIs.

From a woman's perspective, the subjective burden of self-perceived illness and abnormality can be as important as a result of disease due to a biomedical confirmed condition. For example, in Muslim communities, vaginal discharge is considered troublesome because it affects the woman's prayer requirements to be "clean" [7]. Self-perceived illness and abnormality also affect health-seeking behaviour, leading often to unnecessary spending on health care and ineffective management [8,9]. In fact, the syndromic management of vaginal discharge can lead to inappropriate treatment of a large proportion of women, as well as unnecessary spending on treatment of non-existing RTIs [8,13].

An alternative explanation to MUVD has been recently suggested. Several observational studies confirmed the contribution of anxiety and depression to medically unexplained gynaecological symptoms, such as pelvic pain and abnormal vaginal discharge [5,12,14-22]. Taking the already discussed UHS, the sub-sample analysis pertaining to Hey el Selloum sample, showed that a large proportion of women (42%) reported mental distress- as assessed by GHQ-12. Moreover, multivariate analysis showed that mental distress was significantly associated with reported abnormal vaginal discharge, after adjusting for relevant risk factors and reported RTIs [23]. Women considered stress to be a main cause of their discharge [24] and identified mental as well as psychological health as important elements contributing to their perception of good reproductive health [4]. The UHS also showed that both mental distress and reported gynaecological health problems were negatively associated with social support [25].

Along the same line, population-based studies from South Asia show significant associations between complaints of vaginal discharge and psychosocial stress, in addition to psychosomatic symptoms such as dizziness, backache, and weakness [12,14,26]. Qualitative studies in India indicated that women typically associate complaints of discharge with mental stress and related symptoms such as tiredness [12]. On the other hand, social support is consistently associated with common mental disorders (CMD) and physical conditions [27]. Improvements in social support were shown to lead to lower consultation rates [27,28] and reduced sickness absences [29].

These findings have important implications for alternative intervention strategies. Our proposed psychosocial intervention carried out in this trial (consisting of relaxing exercise and social support) aims to reduce MUVD as a result of reducing CMDs. There are only a few randomized controlled trials (RCTs) for the treatment of CMDs in the developing world [30-33]. Most of these studies have used traditional psychological models such as Cognitive Behavioural Therapy and/or Interpersonal Therapy to alleviate CMDs [34,35]. However, some studies in the West have shown the effectiveness of simpler alternative approaches such as problem solving [36,37] and group support strategies [30,38-43]. Similarly, different RCTs have demonstrated that progressive muscle relaxation and/or guided imagery can alleviate depressive symptoms by enhancing self efficacy and decreasing persistent unexplained physical symptoms [43-47].

We conducted a community-based randomized trial, comparing the experimental group (who were administered the psychosocial intervention) with the control group (who served as a treat later group). The research question is the following: **Does a community based psycho-social intervention **(combined structured social support groups and progressive relaxing exercises) alter **complaints from medically unexplained vaginal discharge **among low-income married women, aged 18-49 and residing in Hey el Sellom?

## Methods/Design

### Design

A community-based randomized trial was conducted to evaluate the impact of a psychosocial intervention on medically unexplained vaginal discharge (MUVD) in a group of married, low-income Lebanese women, aged 18-49, and suffering from low to moderate levels of common mental disorders (CMD) (anxiety and/or depression). The trial consisted of two arms: (a) Women in the experimental group who received psychosocial intervention, (b) women in the control group (treat later). The institutional review board of the American University of Beirut approved the study protocol.

### Setting & Study Population

The trial took place in Hey el Sellom, a neighbourhood located in the southern suburbs of Beirut, Lebanon. It is a large informal settlement of an estimated population of 150,000 (mainly Lebanese Shiites). The area suffers from official neglect and lacks basic health, education and physical infrastructure. Data from the UHS show that up to 60 per cent of inhabitants consider themselves 'poor'. Women have low education, with about 1 out of 4 women only reaching elementary school (1).

#### Study Inclusion Criteria

- Currently married women, 18-49 years of age

- Women who reported symptoms of vaginal discharge confirmed by medical exam to be MUVD

- Women who scored low to moderate levels of Common Mental Disorders (CMDs), based on the study screening tool for common mental disorders (Hopkins Checklist 25)

- Women who were willing to take part in the study by signing an informed consent after listening to our explanation of the study process

- Women who upon screening had none of the exclusion criteria listed below

#### Study Exclusion Criteria

- Pregnancy

- Menopause

- Less than 8 weeks postpartum

- Hysterectomy

- Women who score very low or severe levels of CMDs,

- Women who report having been treated for severe mental illness such as bipolar depression, schizophrenia and suicidal plans

### Sample Size

The sample size calculation was based on the primary outcome of reported MUVD at six months. Allowing for 10% attrition, a sample size of 80-102 in each group would provide 80-90% power to detect a difference of 20 percentage points (for example, 10% vs. 30%) in the proportion reporting MUVD at 6 months, using a 2-sided 5% significance level. There is no previous study that can directly inform the effect size likely for this outcome, but studies have found differences in recovery rates for CMD of up to 40% between usual care and active interventions for common mental disorders (4, 10, 11). We have opted for a 20 percentage point difference given these previous studies on CMD and our judgement that this is both a clinically significant and plausible difference in MUVD rates.

### Recruitment

Sample recruitment was facilitated by adopting a community-based participatory research approach. The recruitment of the women, the medical assessments, the data collection and the delivery of the intervention package was facilitated by our trial stakeholders: the Ministry of Social Affairs and Amel association. It was also facilitated by the assistance and support of the available schools, factories, gynaecological clinics, and satellite network providers found in the area. It was ensured that the different community counterparts were evenly distributed throughout the 11 different community neighbourhoods of Hey el Selloum. During recruitment, a baseline questionnaire was administered to determine whether or not the women initially met the trial's inclusion criteria. Women reporting vaginal discharge were referred to undertake a pelvic as well as laboratory check up- to rule out the presence of RTIs (following informed consent). Women who did not report any vaginal discharge were referred for a general physical exam, unless they insisted to undertake the pelvic and laboratory checkups.

### Randomization and Procedure

Randomization was performed using a computer-generated allocation schedule, produced by an individual not involved in the recruitment process. The original protocol was that the baseline measures and consent were planned to be obtained before allocation was determined remotely (by telephone) and then revealed to the woman. In the event, logistical constraints at the recruitment centres meant that remote allocation was not feasible and allocation was performed using the same computerized system as originally intended, but in the field rather than remotely. An unplanned consequence of this change in procedure was that the women were informed of their allocation before they gave written consent.

The women were informed of their allocation into either the intervention or the control arm (treat later and given the intervention after the 6 months follow up) when they returned to pick up their laboratory results. Women who did not obtain their results were contacted by telephone, up to a maximum of three times.

Blinding on the intervention status was not possible for both logistic and ethical reasons, especially as knowledge of involvement in the intervention was integral to its delivery.

### Intervention

The intervention package was given for six weeks. The package combined trainer-supervised relaxation exercises (twice per week for 30 minutes/session) and psycho-educational group discussions (twice per week for 75 minutes/session). The order of each session was determined by practical circumstances. The trainer-supervised relaxation exercises consisted of progressive muscle relaxation, guided imagery, stretching and breathing, and progressive resistance exercise. The psycho-educational sessions were divided into directed and semi-structured social support discussion sessions, incorporating problem solving skills building as well as venting. The relaxation exercises were given, over six weeks by trained physical fitness instructors, and the group discussions were given by clinical psychologists and assisted by co-moderates who were social workers. The fitness instructors were trained prior to the intervention by a specialist in muscle relaxation and guide imagery. Similarly, the clinical psychologists received two days of training from a senior clinical psychologist, and were provided with a manual that describes in detail each of the 12 sessions. To ensure coherence among group members, once a group was formed its members remained in the same group throughout the intervention period. The benefits to women enrolled in the trial included: the provision of free childcare during sessions, free pelvic exam, and free laboratory tests.

No major risks were anticipated for the participating women in this trial. There were potentially some discomforting issues, such as repeated pelvic exam and laboratory tests at baseline and at six months, an active commitment to attend sessions throughout the 6 weeks intervention periods, as well as answering some sensitive questions during the interview questionnaires. On the other hand, the control group were provided with a periodic mental health assessment during intervention delivery, free pelvic exam and laboratory tests at baseline and six months.

In particular, for the control (treat later) group, two clinical psychologists monitored them closely (every three weeks) by assessing their depression/suicidal status via administering the Arabic validated Hopkins Checklist-25 (depression subscale, which is comprised of 15 questions to measure depression coupled with an additional question on any plans to commit suicide during the past week). This was perceived to be more ethical to ensure that their CMD status did not worsen during the intervention period for the experimental group. All women with severe depression or who reported plans to commit suicide (whether in the intervention or control group) were referred for free follow-up visits at a community psychiatric clinic, belonging to Medicins Sans Frontier and located in close proximity to Hey el Selloum, in a neighbourhood called Burj el Barajneh.

As for the intervention package, it should be noted that the components and the duration of the intervention package were supported by evidence-based literature findings pertaining to the positive impact of structured social support on disease [48-57] and mental health outcomes [30,38-43,55-57]. The literature also indicates the positive impacts of progressive muscle relaxation and/or guided imagery on mental health and persistent unexplained physical symptoms [44-47].

It should be noted that the particular use of this psychosocial intervention, as opposed to other more sophisticated psychological based methods (cognitive behavioural therapy [CBT] or interpersonal therapy [IPT]), was motivated by the fact that it requires less training and is perceived to be more culturally acceptable, in a conservative-economically disadvantaged Muslim community. The other methods require a larger number of well-trained experts as well as longer training periods before actual implementation, which is beyond the capacities of the trial community. The overarching aim of this trial was to adopt a simple low cost community-based approach (that requires moderate levels of education and experience), with the hope to be later taken over (if proved effective) by the local community centres. It should be noted that the different intervention components were pilot-tested for adequacy, appropriateness and cultural acceptability before actual implementation.

The semi-structured support (SSS) sessions were designed to be partially directed, with semi-structured components. They also incorporated trainings on problem solving and social support. The aim behind this combination was to enable women to participate in the discussion as well as to build rapport. The main desired outcomes of the SSS components were: empathy, venting and problem solving.

The relaxation exercise component of the intervention aimed to train the women on how to engage in guided imagery exercises in their own homes. It was unlikely considered that if the women replicate some of the physical fitness components at home or inform other women about these components (for example some women in the control group) this would result in contamination. Besides, the relaxation exercise sessions were progressive in nature, i.e. did not follow the same routine, starting at 15 minutes and building up resistance to reach 30 minutes. This also contributes to a lower probability of contamination.

Each new session of the relaxation exercise package was designed to wrap up the techniques conducted in the previous sessions, touch upon what is being practiced at home and proceed by adding an additional new technique.

Attendance was continuously monitored at the beginning of each session (SSS and relaxation exercise). A checklist was also used to indicate the techniques learnt in sessions, techniques practised at home, etc.

### Instruments

The primary outcome is MUVD at six months. A woman is considered to have MUVD, if she reports a complaint of vaginal discharge as assessed by the question:" are you complaining currently from vaginal discharge?" and is concurrently not suffering from any RTIs, as confirmed by pelvic exam and laboratory tests (using swabs). Further questions were also asked of the women to note whether or not they are bothered by MUVD, as well as to indicate the colour, odour, thickness, consistency and frequency of discharge. Besides, relevant health service use such as consultations for vaginal discharge and associated treatments were also explored.

#### The secondary outcomes include

- CMD: the Hopkins Symptom Checklist 25 (HSCL-25) (individual scores on anxiety and/or depression > 1.99 are considered symptomatic was used to assess common mental disorders). The HSCL-25 is known to have good psychometric measures [58-61]. This study instrument was field tested for validity and internal consistency on a convenience sample of 153 currently married women, aged 18-49, living in similar conditions in a different area (Ma3amourrah) of the study target group using the MINI International Neuropsychiatric Interview to serve as the diagnostic criteria. For HSCL-25 depression, we recommend the use of 2.10 as the cut off value. This cut off value showed a good balance between sensitivity and specificity, NPV and PPV. It also had the highest kappa value (0.47) among all the other cut offs. As for the HSCL-25 anxiety subscale, the cut off value of choice was 2.00. This value showed a good balance among the various measures and the highest kappa value. The Mini International Neuropsychiatric Interview (MINI) was chosen as the gold standard since it has been validated in Arabic against the Composite International Diagnostic Interview (CIDI) [62]. It has also been validated in Moroccan colloquial Arabic using expert diagnosis by Kadri et al in 2005 [63]. Both validation processes showed that MINI demonstrated good psychometric properties.

- Somatisation: assessed using The Scale for Assessment of Somatic Symptoms (SASS) - the Patel Somatisation Scale, 2005. This scale has been shown to have good psychometric measures in Asian countries [17]. It measures four clusters of somatic symptoms: pain related symptoms (5 questions), sensory somatic symptoms (5 questions), non specific somatic symptoms (5 questions), biological function related symptoms (5 questions). For the purpose of our trial, it was validated by face validity using the expertise of mental health experts.

#### Other measures/cofactors included

- Socio-demographic factors: age, education, employment status, family composition (presence of extended family members), number of children present (by age of each child), housing tenure (owned or rental), number of rooms, and household income.

- Women's reproductive and obstetric history (e.g. pregnancies, live births, abortions, still births).

- Social Support: assessed using questions from the Urban Health Survey, already translated into Arabic.

#### Timing of assessments

Data were collected at the screening phase, at 1.5 months and at six months, with MUVD assessed at baseline and six months. CMDs were assessed at screening, at 1.5 and at six months after randomisation, and somatisation was assessed at baseline and at six months. The laboratory tests to rule out RTIs were assessed at baseline and at six months. The socio-demographic characteristics and reproductive history of the women were assessed only at baseline, while, social support factors were assessed at both baseline and at six months.

### Analyses

The trial will be analyzed and reported in accordance with the CONSORT guidelines. An interim analysis was not planned for this trial. Descriptive statistics will be used to compare the two groups as randomized. The primary analysis will comprise the difference in the percentages of women with MUVD at 6 months between the groups as randomized (including the 95% confidence interval) and the number-needed-to-treat. Logistic regression analyses (for the primary and secondary outcomes) will be used to adjust for any baseline imbalances, and imputation methods will be used to replace missing outcome data. Clustering effects of the intervention groups will be accounted for using methods such as robust standard errors and mixed effects regression models. Potential mediators will be investigated by adding (separately) changes in HSCL-25 Anxiety and Depression sub-scales and the somatisation scale scores to the regression models with the primary outcome. Adherence to the intervention will be accounted for using instrumental variables regression models. Appropriate interaction terms will be added to the regression models for the primary and secondary outcomes to investigate differential effects of the intervention according to age and baseline HSCL-25 scores, all as continuous variables.

## Discussion

The approach adopted for the randomized trial ensured the involvement of the community throughout the different stages of the trial: preparation, recruitment, training on intervention and data collection, as well data analysis, data reporting and dissemination. Two committees that are representative of the women in the community of Hey el Selloum were formed. The purpose of these two committees was to ensure that woman's voices (specifically are the grass root level) are being heard, in order to: better understand their language, better service to their needs, as well as understand the cultural context. These two committees were the Local Women Committee (LWC) and the Community Advisory Board (CAB). The LWC was composed of representative women from the community with good outreach experience and sound interaction with the local women in Hey el Selloum. The LWC meets regularly on a weekly basis. The CAB, on the other hand, was composed of two representatives from the different community stakeholders who are involved in this trial. The CAB served as the supervisory/monitoring liaison between the technical research team represented by the steering committee and the community. The CAB met on a bi-weekly/monthly basis or as deemed necessary during preparatory phase and the intervention delivery periods. These two committees were established following a comprehensive community mapping, establishing contacts with all stakeholders, qualitative interviews with men and women and focus groups with community midwives in order to assess the trial's as well as the intervention's feasibility.

The study results will be disseminated to the public once the analysis of the trial is completed. Results' dissemination will be at three different levels: the CAB, the LWC and the community at large. The language and degree of complexity of the dissemination efforts will take into account the educational level, cultural issues, and inherent social characteristics of the receiving audience.

This trial presents a novel intervention strategy in reproductive as well as mental health research. The trial aims to assess the impact of a simple, low cost, culturally appropriate psychosocial intervention on alleviating the symptoms of reported MUVD, among a group of low-income Lebanese married women aged 18-49, residing in Hay el Selloum (a southern disadvantaged suburb in Beirut, Lebanon), suffering from low-moderate levels of common mental distress (anxiety and/or depression). The intervention was a six-weeks (twice per week) package, consisting of relaxation exercises and social support.

The proposed intervention package also seemed more appropriate for a setting such as Lebanon, given the limited availability of adequately trained psychotherapists, limited financial resources coupled with the high rates of CMDs in the country (in light of lack of a national-based insurance coverage for such conditions, and abuse of over the counter tranquilizers and sedatives) [64-66].

## Ethical approval

This trial gained ethical approval by the Institutional Review Board of the American University of Beirut.

## Competing interests

The authors declare that they have no competing interests.

## Authors' contributions

All authors have contributed to, read and approved this manuscript.

## Pre-publication history

The pre-publication history for this paper can be accessed here:

http://www.biomedcentral.com/1471-244X/11/142/prepub
